# Retinol-binding protein 3 in ophthalmology: current evidence, research progress, and future perspectives

**DOI:** 10.3389/fimmu.2026.1865865

**Published:** 2026-07-13

**Authors:** Meilin Li, Rongbin Liang, Shijin Guo, Xiaodong Zhou

**Affiliations:** 1 Department of Ophthalmology, Jinshan Hospital of Fudan University, Shanghai, China; 2 Department of Ophthalmology, Eye & ENT Hospital of Fudan University, Shanghai, China; 3 Shanghai Key Laboratory of Visual Impairment and Restoration, Fudan University, Shanghai, China; 4 Department of Orthopedics, Jinshan Hospital of Fudan University, Shanghai, China

**Keywords:** retinol-binding protein 3, visual cycle, photoreceptor function, diabetic retinopathy, retinal diseases

## Abstract

Retinol-binding protein 3 (RBP3) is a photoreceptor-derived glyco-lipoprotein that plays a central role in retinoid trafficking between photoreceptors and the retinal pigment epithelium. Beyond its canonical role in the visual cycle, emerging evidence suggests that RBP3 may participate in retinal metabolic regulation, neurovascular protection, inflammatory modulation, and disease-related immune responses. This review critically summarizes current evidence regarding RBP3 in diabetic retinopathy, retinal degeneration, pathological myopia, primary congenital glaucoma, and uveitis. Among these conditions, diabetic retinopathy has the most extensive evidence base, including clinical association studies, proteomic analyses, and preclinical intervention experiments. However, most clinical studies remain correlational, and RBP3-based therapeutic strategies have not yet been validated in human interventional trials. In other diseases, such as primary congenital glaucoma and uveitis, the current evidence is more limited and is largely derived from proteomics or animal models. Therefore, RBP3 should currently be regarded as a promising but incompletely validated biomarker and therapeutic candidate. Future studies should clarify disease-stage-specific roles, receptor-mediated mechanisms, safety profiles, and clinical utility through large-scale prospective and mechanistic studies.

## 1 Introduction

Retinol-binding protein 3 (RBP3), also known as interphotoreceptor retinoid-binding protein (IRBP), is primarily localized in the interphotoreceptor matrix (IPM), a specialized extracellular compartment located between the external limiting membrane of the retina and the apical surface of the retinal pigment epithelium (RPE). The IPM surrounds the inner and outer segments of photoreceptors that extend from the outer retinal surface. RBP3 is a large glyco-lipoprotein with a molecular weight of approximately 143 kDa. It consists of four homologous domains, each containing approximately 300 amino acids, and is predominantly secreted by rod and cone photoreceptors. Owing to its large molecular size, which exceeds the size limit imposed by the zonulae adherens between photoreceptors and Müller cells, RBP3 is effectively retained within the IPM (1, 2).

RBP3 mediates the transport of retinoids, a class of vitamin A derivatives including retinol and retinal, between photoreceptors and the RPE. Specifically, RBP3 facilitates the targeted delivery of all-trans-retinol to the RPE, promotes the release of 11-cis-retinal from the RPE, and enhances the release of all-trans-retinol from rod outer segments (3). During this transport process, RBP3 protects these labile molecules from oxidation and spontaneous isomerization (4). In recent years, increasing evidence has suggested that RBP3 is not only a key regulator of the visual cycle but may also exert protective effects or participate in pathological processes in various ocular diseases.

## 2 Literature search strategy

A targeted PubMed search was performed to identify relevant studies for this narrative critical review. The search covered publications available up to May 2026. Search terms included “RBP3”, “IRBP”, “interphotoreceptor retinoid-binding protein”, “retinol-binding protein 3”, “visual cycle”, “diabetic retinopathy”, “retinal degeneration”, “retinitis pigmentosa”, “pathological myopia”, “primary congenital glaucoma”, “uveitis”, and “experimental autoimmune uveitis”, either alone or in combination with disease- or mechanism-related terms. Additional relevant articles were identified by manually screening the reference lists of selected publications and related reviews. Original research articles, clinical association studies, proteomic studies, genetic studies, animal experiments, *in vitro* mechanistic studies, and relevant reviews were considered. Studies were excluded if they were not directly related to RBP3 or ocular disease, or lacked original mechanistic or clinical information relevant to the scope of this review. Because this was a narrative critical review rather than a systematic review, no formal risk-of-bias scoring or quantitative evidence grading was performed, no quantitative meta-analysis was performed. Instead, the strength of evidence was qualitatively assessed according to study type, disease context, experimental model, and degree of clinical validation.

## 3 Aim and scope of this review

This article is designed as a narrative critical review rather than a systematic review. We aimed to summarize and critically evaluate current evidence on the physiological functions and disease-related roles of RBP3 in ophthalmology, with particular emphasis on the strength and limitations of available evidence. The review focuses on five major disease contexts: diabetic retinopathy, retinal degeneration, pathological myopia, primary congenital glaucoma, and uveitis. Because the available literature on RBP3 is unevenly distributed across ocular diseases, the length and depth of each disease section in this review are evidence-weighted rather than uniformly allocated. Diabetic retinopathy is discussed in greater detail because it currently has the most comprehensive evidence base, including clinical association studies, intraocular proteomics, animal intervention studies, and *in vitro* mechanistic work. In contrast, the evidence for primary congenital glaucoma and uveitis is more limited and mainly consists of proteomic associations or experimental autoimmune uveitis models. Accordingly, these sections are presented more cautiously as hypothesis-generating or model-based evidence.

To avoid overinterpretation, the evidence discussed in this review is categorized conceptually into four levels: established physiological functions, plausible mechanistic hypotheses, preclinical therapeutic observations, and speculative translational prospects. The role of RBP3 in retinoid transport and visual cycle homeostasis is well established. In contrast, its disease-modifying effects, biomarker utility, and therapeutic application remain incompletely validated and should be interpreted according to disease context and evidence type.

## 4 Physiological functions and expression regulation of RBP3

### 4.1 Physiological functions of RBP3

The visual cycle, also known as the retinoid cycle, comprises a series of enzymatic reactions initiated by light-induced activation of visual pigments in photoreceptors. Its central function is the continuous regeneration of 11-cis-retinal to replenish chromophores consumed by light activation, thereby maintaining photoreceptor light sensitivity (5, 6). In the canonical RPE-dependent visual cycle, 11-cis-retinal is synthesized in the RPE and supplied to both rods and cones, whereas additional cone-related retinoid cycling pathways involving Müller cells have also been described (7, 8).

Following photoactivation of visual pigments, 11-cis-retinal is photoisomerized to all-trans-retinal and released from opsins, including rhodopsin in rods and cone opsins in cones. Subsequently, retinol dehydrogenases (RDHs) catalyze the reduction of all-trans-retinal to all-trans-retinol in photoreceptors (9, 10). A portion of free all-trans-retinal may also be released into the disk lumen.

**Figure 1:**
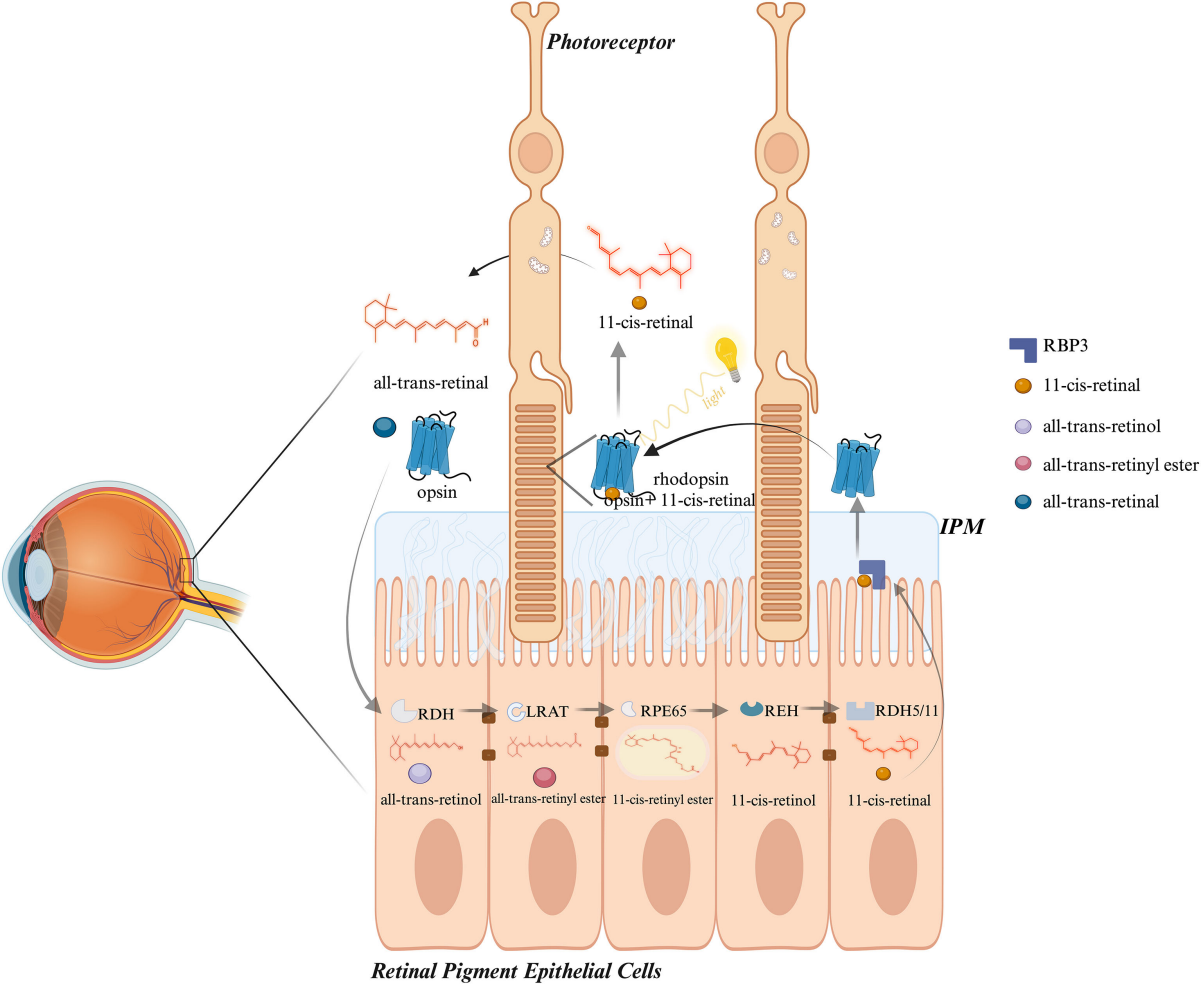
Role of RBP3 in the visual cycle. In photoreceptors, 11-cis-retinal binds to opsin to form rhodopsin. Upon light exposure, 11-cis-retinal is photoisomerized to all-trans-retinal, which is subsequently reduced to all-trans-retinol by retinol dehydrogenase. RBP3 transports retinoids through the IPM between photoreceptors and the RPE. In RPE cells, all-trans-retinol is esterified by LRAT to all-trans-retinyl ester, converted by RPE65 and related enzymes to 11-cis-retinol, and then oxidized by RDH5/11 to 11-cis-retinal. The regenerated 11-cis-retinal is transported back to photoreceptors to sustain the visual cycle. RBP3, retinol-binding protein 3; IPM, interphotoreceptor matrix; RDH, retinol dehydrogenase; LRAT, lecithin,retinol acyltransferase; RPE65, RPE-specific 65 kDa protein; REH, retinyl ester hydrolase; RDH5/11, retinol dehydrogenase 5/11.

All-trans-retinol is then transported through the IPM to the RPE with the assistance of RBP3 (11). In the RPE, all-trans-retinol is esterified by lecithin:retinol acyltransferase (LRAT) to generate all-trans-retinyl esters. These esters may be stored in retinyl ester storage particles (12), also known as retinosomes, or used as substrates by RPE65 to produce 11-cis-retinol (13–15). 11-cis-retinol is subsequently oxidized by RDH5, RDH11, and other RDH enzymes to generate 11-cis-retinal. The newly synthesized 11-cis-retinal then diffuses back to the outer segments of rods and cones, where it recombines with opsins to regenerate rhodopsin and cone pigments, thereby completing the visual cycle (16). The overall role of RBP3 in the visual cycle is illustrated in **Figure 1**.

RBP3 contains four homologous retinoid-binding modules, with reported binding affinities in the following order: 11-cis-retinal > all-trans-retinol > all-trans-retinal > 11-cis-retinol (17, 18). Within the visual cycle, RBP3 binds and stabilizes all-trans-retinol, thereby protecting it from photodegradation and spontaneous isomerization in the IPM. This function ensures efficient and safe retinoid transport between photoreceptors and the RPE and makes RBP3 one of the key extracellular carriers required for the proper operation of the visual cycle. In addition, RBP3 promotes the release of all-trans-retinol from bleached rod photoreceptors and facilitates the release of 11-cis-retinal from the RPE. *In vitro* studies have further shown that RBP3 promotes all-trans-retinol uptake and 11-cis-retinol release by Müller cells, suggesting its potential involvement in the cone visual cycle (19, 20).

RBP3 also removes retinol and retinal from light-activated rod outer segments (21), thereby inhibiting the formation of light-induced lipofuscin precursors, although it does not remove pre-existing precursors (11). By accelerating rhodopsin regeneration and indirectly reducing reactive oxygen species (ROS) generated by photic injury, RBP3 may protect the retina from photo-oxidative damage (3, 22). Beyond its retinoid-binding capacity, RBP3 can also bind long-chain fatty acids, including oleic acid and docosahexaenoic acid (23, 24). These lipid interactions may facilitate the movement of fatty acids across the IPM and dynamically modulate the affinity of RBP3 for different retinoid isomers. The type of fatty acid bound to RBP3 may influence its retinoid-binding and release properties, suggesting additional functions and regulatory mechanisms in retinoid delivery and uptake (25, 26).

In addition to its role in retinoid transport, RBP3 plays an important role in retinal development and homeostasis. Its expression begins early during retinal differentiation, preceding the establishment of the mature visual cycle in developing mice (27, 28). Cysteine residues within RBP3 may contribute to the maintenance of redox balance in the healthy retina (22). Studies have shown that RBP3 deficiency leads to abnormal axial elongation and thinning of the outer nuclear layer from early ocular development in mice, suggesting that RBP3 is essential for early eye growth and the maintenance of normal refractive status (29). Clinically, homozygous mutations in RBP3 have been reported in children with retinal dystrophy, and RBP3 mutations represent a rare cause of autosomal recessive retinitis pigmentosa (RP) (30). These findings support the indispensable role of RBP3 in normal ocular development and visual function (31, 32).

### 4.2 Expression sites and regulatory mechanisms of RBP3

RBP3 expression is largely restricted to photoreceptors and the pineal gland, whereas low levels of the protein can also be detected in ocular fluids such as the vitreous and aqueous humor. RBP3 is found only in vertebrates, and acquisition of the RBP3 gene during vertebrate evolution may have contributed to the development of a more complex visual retinoid cycle (33). RBP3 is primarily an extracellular protein (34). However, a fraction of RBP3 has also been detected in RPE phagosomes and endocytic vesicles, and the amount of RBP3 within the RPE may reach approximately 20% of the total amount present in the IPM.

The distribution of RBP3 within the IPM is regulated by light and dark adaptation (35). Under light-adapted conditions, RBP3 is mainly associated with the RPE and photoreceptor cell membranes, whereas under dark-adapted conditions it exhibits a more uniform distribution throughout the IPM (36). RBP3 accounts for approximately 5% of the soluble protein content of the IPM and is important for maintaining the structural integrity of this extracellular compartment. RBP3 may interact with the hyaluronan scaffold of the extracellular matrix, which forms non-covalent complexes with proteoglycans (37). Therefore, RBP3 dysregulation may disrupt IPM organization and retinoid homeostasis, thereby contributing to the development of multiple retinal diseases.

The expression of RBP3 in photoreceptors is regulated, at least in part, by the cone-rod homeobox transcription factor Cone-Rod Homeobox (CRX), a photoreceptor-associated homeodomain transcription factor that is essential for photoreceptor differentiation and maintenance; CRX can bind and transactivate photoreceptor-specific regulatory elements, and CRX-binding elements have been identified in the RBP3 promoter region, supporting a direct role for CRX in RBP3 transcriptional regulation (38–40). In zebrafish retina, RBP3 mRNA levels are regulated by circadian rhythm, whereas RBP3 protein levels remain relatively constant throughout the day-night cycle (41). Increased daytime mRNA expression may compensate for more rapid protein turnover. It should be noted that these circadian observations were obtained under physiological conditions and should not be directly extrapolated to pathological states. Whether diabetes or retinal inflammation disrupts the normal relationship between RBP3 transcription, secretion, and protein stability remains unknown and requires direct investigation.

### 4.3 Molecular structure and ligand-binding mechanisms of RBP3

RBP3 is a highly conserved large glyco-lipoprotein. Its primary structure consists of four tandem homologous domains, each containing approximately 300 amino acids, suggesting that it may have originated from ancient gene duplication events and acquired the ability to bind multiple ligands. Previous studies indicate that RBP3 does not simply mediate passive diffusion of retinoids. Rather, it selectively binds different retinoid isomers and lipid ligands through multiple hydrophobic binding sites, enabling directional, stable, and relatively protected retinoid transport between photoreceptors and the RPE (42, 43).

*In vitro* binding studies have shown that RBP3 can bind multiple visual-cycle intermediates, including all-trans-retinol, 11-cis-retinal, all-trans-retinal, and 11-cis-retinol. Its differential affinity for these retinoids may help maintain the directionality of ligand loading, transport, and release during the visual cycle. In addition to retinoids, RBP3 can bind long-chain fatty acids, particularly unsaturated fatty acids. Ghosh et al. reported that the crystal structure of zebrafish RBP3 revealed a deep hydrophobic pocket within the N-terminal domain A capable of binding oleic acid (24). Fluorescence titration experiments further showed that oleic acid could displace all-trans-retinol bound to RBP3, suggesting that fatty acids may regulate the retinoid-binding capacity of RBP3 by altering protein conformation or competing for binding sites.

More recently, Kaushik et al. used cryo-electron microscopy and small-angle X-ray scattering to resolve the overall structure of porcine RBP3, further confirming that RBP3 is composed of multiple retinoid-binding modules and suggesting that the protein exhibits conformational flexibility in solution. This structural feature may facilitate ligand loading and release at different cellular interfaces, including photoreceptors, Müller cells, and the RPE (18, 43). Taken together, these findings indicate that RBP3 is not merely a “retinoid carrier” but rather a dynamic transport platform regulated by lipid ligands, the local microenvironment, and protein conformation. Structural abnormalities or impaired ligand-binding capacity of RBP3 may disrupt retinoid homeostasis, promote the accumulation of lipofuscin precursors, increase photo-oxidative damage to photoreceptors, and contribute to retinal degeneration.

## 5 Disease-specific evidence for RBP3 in ophthalmology

Experimental and animal studies suggest that altered RBP3 expression may occur early in some retinal disease models. These findings have raised the possibility that RBP3 could serve as an early disease-associated marker in selected contexts, although its predictive value has not yet been validated across different retinal diseases or in large prospective clinical cohorts. Abnormal RBP3 function has been implicated in the pathogenesis of several retinal diseases (44, 45), including diabetic retinopathy (DR),RP, and pathological myopia (PM) (46). RBP3 deficiency has also been shown to lead to abnormal axial elongation of the eye and retinal dysfunction in mouse models (47). This section will systematically explore the research progress on RBP3’s role in various ocular diseases from the disease perspective. A summary of the current evidence for the role of RBP3 across different ophthalmic diseases is provided in **Table 1**.

**Table 1:** Disease-specific evidence for RBP3 in ophthalmology.

Disease	Sample/Source	RBP3 alteration	Key findings	Proposed role or mechanism	Translational Maturity	Evidence level	References
DR	Human vitreous/aqueous, diabetic rat models	Higher in mild/no DR; lower in advanced DR	rhRBP3 reduces vascular leakage, improves ERG, reduces TNF-α/IL-6	Neurovascular protection, VEGF modulation, metabolic regulation, anti-inflammatory effects	Preclinical therapeutic evidence available; no completed human interventional trials	Moderate to relatively strong; supported by clinical association studies, proteomics, animal experiments, and *in vitro* mechanistic studies	(56, 57, 59) etc.
Retinal degeneration/RP	Human genetic studies, RBP3 mutation reports, knockout mice	Loss-of-function or mutation	Photoreceptor degeneration, ER stress, necroptosis, impaired retinoid transport	Impaired retinoid transport, photoreceptor degeneration, ER stress, necroptosis	Mechanistic and genetic evidence; no disease-targeted therapeutic strategy established	Moderate for genetic association; mechanisms require mutation-specific validation	(64, 66) etc.
PM	Knockout mice, GWAS, high myopia families	RBP3 deficiency or mutation associated with axial elongation or high myopia	Axial elongation, high myopia	Altered retinal signaling, visual cycle disturbance, abnormal ocular growth	Early exploratory stage; no therapeutic translation	Low to moderate; based mainly on knockout mice, GWAS associations, and limited familial reports	(29, 47) etc.
PCG	Aqueous humor proteomics	Reduced aqueous RBP3	Altered vitamin A-related pathways; potential effect on aqueous microenvironment	Possible alteration in vitamin A-related pathways or aqueous microenvironment	Exploratory biomarker stage only	Low; proteomic association only	(74, 75) etc.
Uveitis/EAU	Mouse EAU models, immunology studies	RBP3 used as autoantigen	Induces antigen-specific T-cell responses, Th1/Th17 driven ocular inflammation	Antigen-specific T-cell activation and autoimmune retinal inflammation	Established experimental model usage; limited direct human translational relevance	Moderate in animal immune models, but limited human validation	(82) etc.

### 5.1 Diabetic retinopathy

DR is a common and highly diabetes-specific neurovascular complication (48). Meta-analyses have reported that approximately one-third of individuals with diabetes show signs of DR, although prevalence varies by region, diabetes type, disease duration, and diagnostic criteria (49). It is strongly associated with an increased risk of fatal systemic vascular complications, such as stroke, coronary heart disease, and heart failure (50). Diabetes disrupts the balance between pro-survival neurotrophic factors and inflammatory mediators, leading to chronic inflammatory responses that adversely affect retinal endothelial cells and neurons (51). This results in increased vascular permeability, capillary non-perfusion (endothelial cell apoptosis), neurodegeneration (neuronal apoptosis), and neovascularization (52).

The Medalist cohort study found that the severity of DR was not significantly associated with blood glucose control, strongly suggesting the existence of retinal protective factors that counteract hyperglycemic toxicity (53, 54). Proteomic analysis of the retina and vitreous from DR patients showed that RBP3 levels were significantly higher in the retina and vitreous of patients with no DR or mild DR compared to those with advanced DR (55, 56). As a key protein in the visual cycle, RBP3 is crucial for light signal perception, and increasing evidence suggests that retinal neuronal dysfunction and vascular abnormalities in DR occur in parallel. In a study conducted in Lewis rats with insulin-deficient diabetes, recombinant human RBP3 (rhRBP3) or bevacizumab (anti-VEGF antibody) was injected into the vitreous. Three days post-injection, the permeability of the blood-retinal barrier was reduced by 64% in rhRBP3-treated diabetic rats and by 53% in bevacizumab-treated rats (p<0.01). TNF-α, IL-6, and Cd11b levels were significantly elevated in diabetic rats, but rhRBP3 treatment reduced these inflammatory markers to levels similar to controls, whereas bevacizumab had no effect. When rhRBP3 was added to the retinas of diabetic rats, the glycolysis rate was reduced compared to non-diabetic controls (57). Moreover, compared to non-diabetic rats, diabetic rats exhibited significantly lower oscillatory potentials (OPs) and B-wave amplitudes in electroretinograms (ERG) (p<0.01), but vitreous injection of rhRBP3 improved these amplitudes in both non-diabetic and diabetic rats. These findings suggest that RBP3 may represent a candidate biomarker for DR progression and a potential therapeutic target for further investigation, although its clinical utility and interventional value remain to be validated (58).

**Figure 2:**
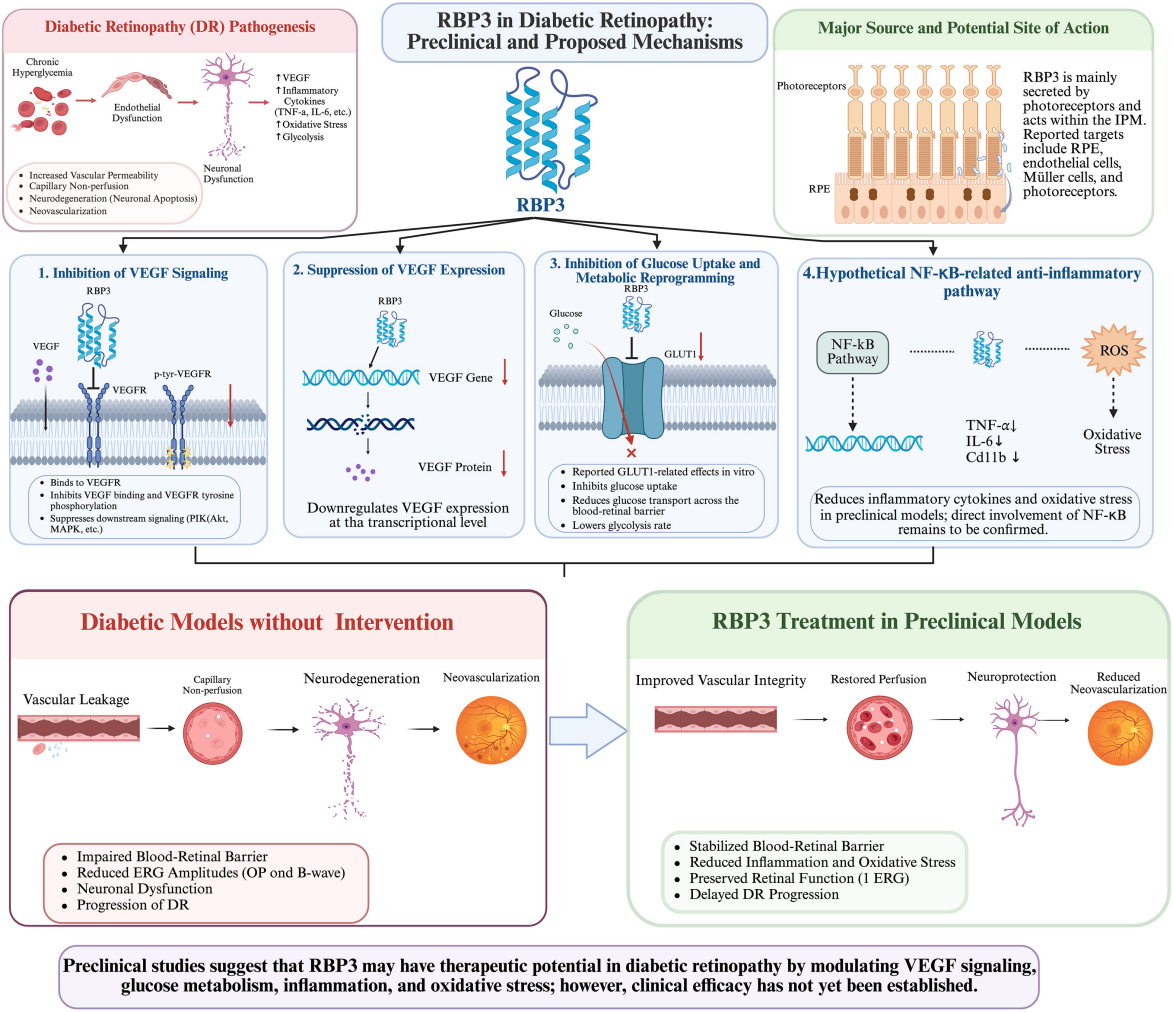
RBP3 in DR: preclinical and proposed mechanisms. Chronic hyperglycemia contributes to DR through increased VEGF signaling, inflammatory cytokine production, oxidative stress, glycolytic activation, endothelial dysfunction, blood-retinal barrier disruption, neurodegeneration, and neovascularization. RBP3 is mainly secreted by photoreceptors and localized in the IPM. Preclinical studies suggest that RBP3 may protect the diabetic retina by interfering with VEGF-VEGFR signaling, reducing VEGF expression, modulating glucose transport and glycolysis, and attenuating inflammation and oxidative stress. However, some pathways, including the involvement of GLUT1 and NF-κB, remain proposed or incompletely established. Solid arrows indicate experimentally supported mechanisms, whereas dashed arrows indicate hypothetical or proposed mechanisms requiring further validation. DR, diabetic retinopathy; VEGF, vascular endothelial growth factor; VEGFR, vascular endothelial growth factor receptor; GLUT1, glucose transporter 1; NF-κB, nuclear factor kappa B; TNF-α, tumor necrosis factor-alpha; IL-6, interleukin-6; ROS, reactive oxygen species; ERG, electroretinography; OPs, oscillatory potentials; PI3K/Akt, phosphoinositide 3-kinase/protein kinase B pathway; MAPK, mitogen-activated protein kinase.

The preclinical effects of RBP3 observed in DR models may involve several mechanisms with different levels of experimental support. First, VEGF/VEGFR-related effects have been reported in experimental settings, including reduced VEGFR tyrosine phosphorylation and decreased hyperglycemia-induced vascular permeability (56, 59). Second, metabolic modulation through GLUT1 has been supported by *in vitro* studies showing that RBP3 can reduce glucose uptake in Müller cells and bovine retinal endothelial cells (55). Third, anti-inflammatory effects, including reductions in TNF-α and IL-6, have been observed in diabetic animal models after rhRBP3 administration. However, it remains unclear whether RBP3 acts through specific receptors or co-receptors, and future research should combine structural biology and protein interaction proteomics to further elucidate its binding spectrum. We hypothesize that RBP3 may indirectly modulate NF-κB-related inflammatory signaling, although direct experimental evidence remains lacking. The proposed mechanisms by which RBP3 may protect against diabetic retinopathy are summarized in **Figure 2**.

As the primary source of RBP3 secretion, the interaction between photoreceptors and the RPE is essential for maintaining photoreceptor structural and functional integrity. The RPE plays a crucial role in transporting water, ions, and nutrients (such as glucose and its metabolites) from the bloodstream to photoreceptors. Therefore, the RPE may represent one important cellular interface for RBP3 activity, supported by evidence of RBP3 internalization by RPE cells (34). However, available experimental evidence also suggests potential effects on photoreceptors, Müller cells, and retinal endothelial cells. Thus, the cellular targets of exogenous or endogenous RBP3 should be interpreted as multiple and context-dependent rather than restricted to the RPE.

Compared with conventional anti-VEGF approaches, preclinical studies suggest that rhRBP3 may affect both vascular and neuroretinal outcomes in diabetic models. In these experimental settings, rhRBP3 was associated with reduced vascular dysfunction, altered glycolytic activity, improved ERG parameters, and decreased inflammatory markers. However, whether RBP3-based treatment provides advantages over anti-VEGF therapy in patients requires direct clinical evaluation. By reducing glycolysis rates, reversing neuroretinal dysfunction, and decreasing diabetes-induced inflammatory factors, RBP3 may represent a candidate strategy for further investigation, particularly for early intervention in DR (57).

Future studies may explore whether RBP3-based approaches could complement existing anti-VEGF therapies, particularly in DR phenotypes characterized by neuroretinal dysfunction, inflammation, or metabolic disturbance. For patients who do not respond well to anti-VEGF therapy or who have significant neuroretinal dysfunction, the possibility of combining or alternating rhRBP3 vitreous injection with anti-VEGF therapy may be explored. However, such combination or alternating strategies remain speculative and have not been tested in human interventional trials. For early or moderate non-proliferative diabetic retinopathy, current animal data suggest that rhRBP3 deserves further investigation as a candidate therapeutic approach, but its efficacy, optimal timing, dosage, durability, and safety remain unknown. Because RBP3 is a recombinant protein, potential immunogenicity and effects on normal retinal metabolism also require careful evaluation. Intravitreal delivery has been explored experimentally, whereas alternative delivery systems, including sustained-release formulations or gene-based approaches, remain future research directions rather than established therapeutic options.

### 5.2 Retinal degeneration

Retinal degeneration refers to a group of diseases caused by various factors, characterized by the progressive degeneration and loss of retinal cells, such as photoreceptors and RPE, which can lead to a progressive decline in vision and even blindness. These diseases include both hereditary conditions (such as RP) and acquired diseases, such as age-related macular degeneration(AMD) and Stargardt disease (60).

RP is a hereditary retinal disease caused by genetic mutations, with progressive degeneration of photoreceptors as its hallmark. Although RP is genetically heterogeneous, rare RBP3 mutations have been reported in patients with inherited retinal dystrophy, indicating that RBP3 dysfunction may contribute to retinal degeneration in selected genetic contexts. The expression of RBP3 in cone and rod cells is regulated by the transcription factor CRX (61), a gene well-established to be associated with retinal dystrophies (62). RBP3 gene knockout (KO) mice exhibit abnormal photoreceptor morphology and a significant reduction in photoreceptor survival, manifesting as early-onset, progressive photoreceptor degeneration (thinning of the outer nuclear layer, loss of rod and cone function), and weakened ERG responses, with these pathological changes progressing slowly over time (63). In a rat model of retinal degeneration induced by bright light, RBP3 mRNA expression significantly decreased after 24 hours of exposure to visible light (490–580 nm, irradiance ~1,200 lux) (64). Studies have shown that RIP3 (Receptor-interacting protein 3) upregulation in RBP3 KO mice promotes programmed necrosis, which accelerates the loss of rod and cone cells. Pharmacological inhibition of RIP3 significantly delays degeneration, suggesting that the inflammatory necrosis pathway is a potential target for intervention (65). A mutation (D1080N) in RP patients, where aspartic acid is replaced by asparagine at position 1080 of RBP3, has been reported (30). This mutation could potentially lead to RP through mechanisms including: (1) mutations associated with RP may impair or abolish the secretory function of RBP3, resulting in loss of its protective roles in nutrient support and retinoid transport; (2) the D1080N mutation may cause RBP3 misfolding, abnormal disulfide bond formation, protein aggregation, and the formation of high-molecular-weight complexes, which cannot be transported to the Golgi apparatus, leading to endoplasmic reticulum stress and cell toxicity (66).

In summary, experimental models and rare human genetic reports support an important role for RBP3 in photoreceptor maintenance and retinal homeostasis. Loss or dysfunction of RBP3 may contribute to photoreceptor degeneration through impaired retinoid transport, cellular stress, and cell-death pathways. Currently, there is no specific therapeutic strategy targeting RBP3-related pathways, making RBP3 and its associated signaling pathways potential targets for intervention in retinal degeneration.

### 5.3 Pathological myopia

According to the standards of the International Myopia Institute, PM refers to a pathological state associated with excessive axial elongation of the eye, leading to structural changes in the posterior segment of the eye, such as posterior scleral staphyloma, myopic maculopathy, and myopic optic neuropathy, resulting in a decline in best-corrected visual acuity. This condition differs from high myopia, which is merely characterized by refractive error (67).

The core feature of PM is excessive axial elongation, and understanding the molecular mechanisms that influences early eye growth is crucial. Studies have reported that RBP3-deficient mice exhibit excessive eye growth during early development (47). RBP3 contributes to retinoid transport and photoreceptor function, and its expression begins early during mouse eye development, before visual experience. Compared to wild-type C57BL/6J controls, RBP3-deficient mice show reduced retinal inner nuclear layer cell death before eye opening but eventually develop severe myopia, indicating that RBP3 may participate in early ocular growth regulation (29). However, the mechanisms linking RBP3 deficiency to axial elongation and human PM remain incompletely understood.

In a meta-analysis of a genome-wide association study (GWAS), RBP3 was found to be associated with an increased risk of axial length elongation and increased corneal curvature, suggesting a link between RBP3 and non-syndromic human myopia (68). Retinal degeneration associated with RBP3 mutations has been reported in three families, with a total of eight patients, all of whom had high myopia. This suggests that RBP3 mutations may be a rare cause of high myopia (30–32). ddPCR analysis of retinal transcriptional expression in RBP3 KO mice at different time points (P5, P40, P213) revealed that multiple genes related to myopia and inflammation (such as C1qa, Gjd2, Sntb1, Vsx2) showed a time-dependent pattern of “early upregulation—mid-stage decline—late-stage recovery or no change,” but it remains unclear whether the inflammatory pathways associated with RBP3 contribute to the initiation of myopia or represent secondary changes (69). This is a potential area for future research. Moreover, RBP3 may have distinct regulatory windows and mechanisms for myopia and degenerative changes (70).

The exact mechanism by which RBP3 gene mutations lead to PM remains unclear. It is hypothesized that RBP3 deficiency disrupts retinol transport, leading to visual cycle disturbances, impaired photoreceptor function, and abnormal axial growth due to abnormal retinal signals. Another potential mechanism is that RBP3 deficiency leads to the accumulation of all-trans-retinol in the IPM, which impedes rhodopsin regeneration, activates necrosis pathways, and induces photoreceptor degeneration. The causal relationship between these processes and scleral remodeling remains to be clarified.

### 5.4 Primary congenital glaucoma

Primary congenital glaucoma (PCG) is an autosomal recessive genetic disorder caused by abnormal development of the anterior chamber angle. This disease manifests in infancy and is characterized by elevated intraocular pressure due to impaired aqueous humor outflow, along with corneal opacity, photophobia, excessive tearing, and buphthalmos (globe enlargement). The molecular etiology of PCG is only partially understood. Mutations in the CYP1B1 gene, which encodes a cytochrome P450 enzyme, are the most common cause of autosomal recessive PCG, but they account for less than one-third of cases in the population, indicating the existence of other pathogenic mechanisms. Studies have shown that the angiopoietin receptor (tunica interna endothelial cell kinase, TEK) is highly expressed in the endothelial cells of Schlemm’s canal, and reduced TEK signaling leads to dysfunction of the aqueous humor outflow pathway, a novel pathogenic mechanism of PCG (71–73). In addition to the above well-supported genetic and developmental mechanisms, there is also evidence suggesting that vitamin A plays a role in anterior segment development, as retinoic acid and other vitamin A derivatives have been shown to be involved in ocular anterior segment tissue development (74).

Proteomic studies have reported reduced RBP3 levels in the aqueous humor of patients with PCG compared with cataract controls (75). Because RBP3 is involved in retinoid transport, this finding raises the possibility that altered vitamin A-related pathways or changes in the aqueous humor microenvironment may be associated with PCG. However, the current evidence is limited to differential protein screening, and no causal relationship between RBP3 reduction and PCG pathogenesis has been established. Therefore, the association between RBP3 and PCG should be considered preliminary and hypothesis-generating.

### 5.5 Uveitis

Uveitis is a group of inflammatory diseases affecting different parts of the eye, categorized by disease course, affected eye, and the main anatomical location, and includes anterior uveitis, intermediate uveitis, posterior uveitis, and panuveitis (76). Globally, uveitis is a major cause of vision loss, ranking as the fifth or sixth leading cause of blindness (77, 78). Unlike other common causes of vision loss, such as cataracts, AMD, and glaucoma, uveitis can affect individuals of all ages, including children.

Importantly, the role of RBP3 in uveitis differs conceptually from its proposed protective role in diabetic or degenerative retinal diseases. In metabolic and degenerative contexts, endogenous RBP3 may contribute to retinoid trafficking, photoreceptor homeostasis, and neurovascular protection. In contrast, in experimental autoimmune uveitis (EAU) models, selected RBP3-derived peptides are used as retinal autoantigens to induce antigen-specific T-cell responses. Therefore, RBP3 should not be uniformly interpreted as intrinsically protective or pathogenic. Rather, its biological role appears to be disease-, compartment-, and stage-dependent, depending on whether intact endogenous RBP3 participates in retinal homeostasis or whether antigenic RBP3 peptides are presented to the immune system under inflammatory conditions.

Non-infectious uveitis is believed to be autoimmune or immune-mediated. EAU is an antigen-specific, CD4^+^ T cell-dependent non-infectious intraocular inflammation model. This model has been widely used to elucidate the pathological mechanisms of disease onset and immune regulation and to identify genetic susceptibility factors. Commonly used RBP3 peptide fragments and S-antigen can induce EAU in mice, both of which are major components of the photoreceptor outer segments and are considered primary targets of autoimmune attack in EAU (79–81). In EAU models, specific RBP3 peptides presented to T cells can induce pathogenic T-cell responses. Some RBP3 peptide fragments are widely used to establish EAU models because of their high pathogenicity and reproducibility in susceptible experimental animals (82). Dendritic cells (CD11c^+^, MHC-II^+^) in the ocular tissues serve as key antigen-presenting cells during the early stages of EAU, presenting RBP3 peptides to naïve T cells and triggering the immune response. The pathogenic response is primarily driven by Th1 (IFN-γ) and Th17 (IL-17) cells, with both cell types jointly determining the intensity of inflammation and tissue damage at different stages of the disease (83, 84). Classic studies have shown that Th17 cells play a pathogenic role in intraocular inflammation. Pertussis toxin (PTX) is commonly used in immune protocols to drive Th1 polarization; however, in highly susceptible B10.RIII mice, a strong Th1 response to RBP3 can occur even in the absence of PTX. Activated macrophages/monocytes infiltrate the ocular tissues and are one of the major effector cells causing tissue damage, amplifying the injury through mediators such as TNF-α, reactive nitrogen, and oxygen free radicals. Clinical manifestations may include vasculitis/perivascular inflammation, exudative retinal detachment, and granuloma formation (85).

This model provides valuable preclinical insight into antigen-specific immune mechanisms, pathogenic immune-cell populations, and effector pathways in intraocular inflammation; however, findings from EAU models should not be directly extrapolated to all forms of human uveitis.

## 6 Discussion

### 6.1 Detection Methods of RBP3 and its feasibility as a biomarker

With advances in proteomics, immunoassays, and extracellular vesicle analysis, RBP3 has attracted increasing attention as a potential biomarker for ocular diseases. Current sample sources for RBP3 detection include aqueous humor, vitreous humor, plasma or serum, plasma-derived small extracellular vesicles(sEVs), and retinal tissue. Among these, aqueous humor has relatively high clinical feasibility because it can be obtained during cataract surgery, anti-VEGF injection, or other intraocular procedures. Recent studies have shown that lower aqueous humor RBP3 levels are associated with diabetic macular edema and DR progression, suggesting its potential value as a biomarker for disease severity and progression risk (86).

Vitreous humor more directly reflects the retinal microenvironment and may better capture local changes in neurovascular injury, inflammation, and VEGF-related pathways. Fickweiler et al. reported that higher vitreous RBP3 levels were associated with lower inflammatory cytokine and VEGF levels, as well as slower DR progression, supporting its role as a local protective biomarker (55). However, vitreous sampling is invasive and therefore unsuitable for routine screening. In contrast, plasma, serum, and plasma-derived sEVs are more suitable for longitudinal monitoring, although whether peripheral RBP3 levels reliably reflect intraocular pathology remains uncertain. A 2025 study found reduced RBP3 levels in vitreous humor and plasma-derived sEVs from patients with proliferative DR, suggesting that extracellular vesicle-derived RBP3 may be a biomarker for vision-threatening DR (87).

Retinal tissue analysis, including Western blotting, immunohistochemistry, immunofluorescence, mass spectrometry-based proteomics, transcriptomics, and spatial omics, is mainly used in animal studies, donor eyes, or post-mortem samples. These methods are valuable for mechanistic studies but have limited clinical applicability. Beyond DR, decreased aqueous humor RBP3 levels have also been reported in patients with PCG, indicating that RBP3 may have biomarker potential in other ocular diseases. However, its diagnostic value outside DR remains preliminary.

Overall, RBP3 detection is still at the stage of biomarker discovery and early translational exploration. Future studies should establish standardized protocols for sample collection, storage, and detection, and should validate its diagnostic sensitivity, specificity, disease-staging value, and complementarity with optical coherence tomography, fundus photography, and ERG in large, multicenter prospective cohorts.

### 6.2 Exploration of the therapeutic potential and overexpression strategies of RBP3

Given the role of RBP3 in the visual cycle and IPM homeostasis, RBP3 has been proposed as a potential therapeutic target for selected retinal diseases. Disruption of RBP3 expression or function may plausibly contribute to retinal dysfunction through impaired retinoid trafficking, altered photoreceptor metabolism, and disturbed retinal homeostasis. However, the therapeutic potential of RBP3 in retinal diseases should be interpreted according to the strength and type of available evidence.

The strongest therapeutic evidence currently comes from preclinical studies in DR. In animal models of DR, vitreous injection of rhRBP3 has been shown to inhibit retinal vascular leakage, reduce VEGF expression, restore ERG function, and exert anti-inflammatory effects by lowering the expression of inflammatory cytokines such as TNF-α and IL-6. Seahorse XF Analyzer-based metabolic analysis further demonstrated that rhRBP3 injection restored retinal glycolysis rates in diabetic rats to normal levels, thereby maintaining retinal metabolic homeostasis. RBP3 targets multiple pathways, including VEGF, and various pro-disease factors, whereas conventional anti-VEGF antibodies only address vascular abnormalities and fail to improve neuroretinal damage and metabolic dysfunction (57). RhRBP3 exhibits dual neurovascular protective effects, simultaneously inhibiting inflammation, restoring metabolism, and improving neural function, thus delaying early-stage DR progression and offering a novel direction for multi-target therapeutic strategies. It shows comprehensive therapeutic potential that differs from monotherapy with anti-VEGF agents, especially for early intervention in DR. These findings support the concept that RBP3 may exert multiple protective effects in preclinical DR models.

In addition to DR, RBP3 dysregulation may also be relevant to other retinal diseases, but the evidence is weaker and more indirect. Mechanistic studies have shown that RBP3 facilitates the removal of all-trans-retinol and all-trans-retinal from photoreceptor outer segments and may thereby reduce the formation of lipofuscin precursors. RBP3 deficiency or dysfunction may increase retinal exposure to all-trans-retinal, a reactive retinoid intermediate capable of inducing oxidative stress and mitochondrial dysfunction, impaired RBP3-mediated retinoid handling may represent a plausible mechanistic link to AMD (11, 88–91). However, this connection remains speculative. Direct evidence demonstrating that RBP3 deficiency drives lipofuscin accumulation or AMD progression in humans is currently insufficient.

Strategies aimed at increasing RBP3 expression also remain at an exploratory stage. Due to the large molecular weight of the RBP3 protein and the lengthy coding sequence, the complete RBP3 transgene expression cassette, after the addition of promoters and poly A elements, may approach or exceed the packaging capacity of conventional adeno-associated virus (AAV) vectors, thus facing technical limitations for traditional AAV-mediated gene supplementation.

Alternative overexpression strategies have been proposed, but each has important limitations, including the following approaches:

1. Adenovirus vectors (ADV) have a relatively large packaging capacity and high transfection efficiency but are highly immunogenic, which may induce strong immune responses in the host (92–94). Therefore, although adenoviral delivery of RBP3 may be useful for short-term experimental overexpression or proof-of-concept studies, its suitability for long-term intraocular RBP3 replacement remains uncertain and would require careful safety optimization;
2. Small activating RNA (saRNA) is a small non-coding RNA of approximately 21 nucleotides that induces gene expression at the transcriptional level, a phenomenon referred to as RNA activation. This mechanism contrasts with the silencing effect of small interfering RNAs (siRNAs) in RNA interference, offering a novel strategy for upregulating target genes (95, 96). A saRNA designed to upregulate the p21 gene has shown therapeutic effects in a rabbit model of proliferative vitreoretinopathy (97), and saRNA has demonstrated therapeutic potential in several diseases, including tumors, cardiovascular disorders, and metabolic diseases (98–100). However, saRNA-mediated RBP3 upregulation has not yet been clinically validated in retinal disease. Major challenges include efficient delivery to retinal target cells, nuclear localization, cell-type specificity, durability of expression, and off-target effects.
3. Lentiviral vectors have also been explored for stable RBP3 expression in animal models. Recent studies have reported stable expression of RBP3 in animal models through subretinal injection of lentiviral vectors over six months (55), but lentiviral vectors are rarely used clinically and are mostly limited to basic research or preclinical stages.

Overall, the therapeutic rationale for RBP3 is biologically plausible and supported by preclinical DR studies, but clinical translation remains uncertain. The current evidence can be summarized as follows: the physiological role of RBP3 in the visual cycle is well established; rhRBP3-mediated protection in DR is supported by preclinical evidence; RBP3 involvement in AMD-related lipofuscin accumulation is hypothetical; and RBP3 overexpression strategies using ADV, saRNA, lentiviral vectors, or other delivery systems remain experimental. Future studies should focus on defining the optimal disease stage, target cell type, delivery route, dosage, duration of expression, immunogenicity, and long-term safety of RBP3-based interventions. RBP3-targeted therapy is promising but remains experimental. Current evidence supports further translational investigation rather than immediate clinical application.

### 6.3 Key scientific issues and research gaps in RBP3-related research

Available evidence suggests that RBP3 may function as an endogenous protective molecule in DR, with higher intraocular levels being associated with milder lesions and slower disease progression (101). Recent findings further indicate that RBP3 may not only participate in local retinal homeostasis but may also be involved in systemic pathological processes through its presence in body fluids and extracellular vesicles (87). Beyond DR, RBP3 deficiency or mutations have been linked to retinal dystrophies and high myopia, whereas RBP3-derived peptides are widely used as model autoantigens in EAU. These observations raise an important unresolved question: how can the same molecule be associated with retinal protection in some contexts while also providing pathogenic antigenic epitopes in autoimmune inflammation?

This apparent duality may reflect differences among intact endogenous RBP3, exogenously administered recombinant RBP3, and RBP3-derived antigenic peptides presented during autoimmune responses (85). Therefore, the biological role of RBP3 is likely to be highly context-dependent, varying according to disease type, disease stage, molecular form, and local immune or metabolic microenvironment. In DR, most clinical studies remain observational, and it is still unclear whether reduced RBP3 levels precede disease progression or occur as a secondary consequence of retinal damage. Although animal studies suggest that exogenous RBP3 has protective effects, the optimal dosage, timing, delivery route, and stage-specific efficacy remain undefined. It is also unknown whether increasing RBP3 levels would benefit all patients with DR or only selected subgroups.

Although recent studies have renewed interest in RBP3 as a biomarker and therapeutic candidate in ophthalmology, the overall number of publications over the past five years remains limited. This scarcity likely reflects practical and technical constraints rather than a lack of biological relevance. RBP3 is primarily produced by photoreceptors and localized in the IPM, which complicates direct assessment in human retinal tissue. Clinically accessible samples, such as aqueous humor, vitreous humor, plasma, and extracellular vesicles, provide valuable but indirect information and require standardized collection and detection methods.

**Figure 3:**
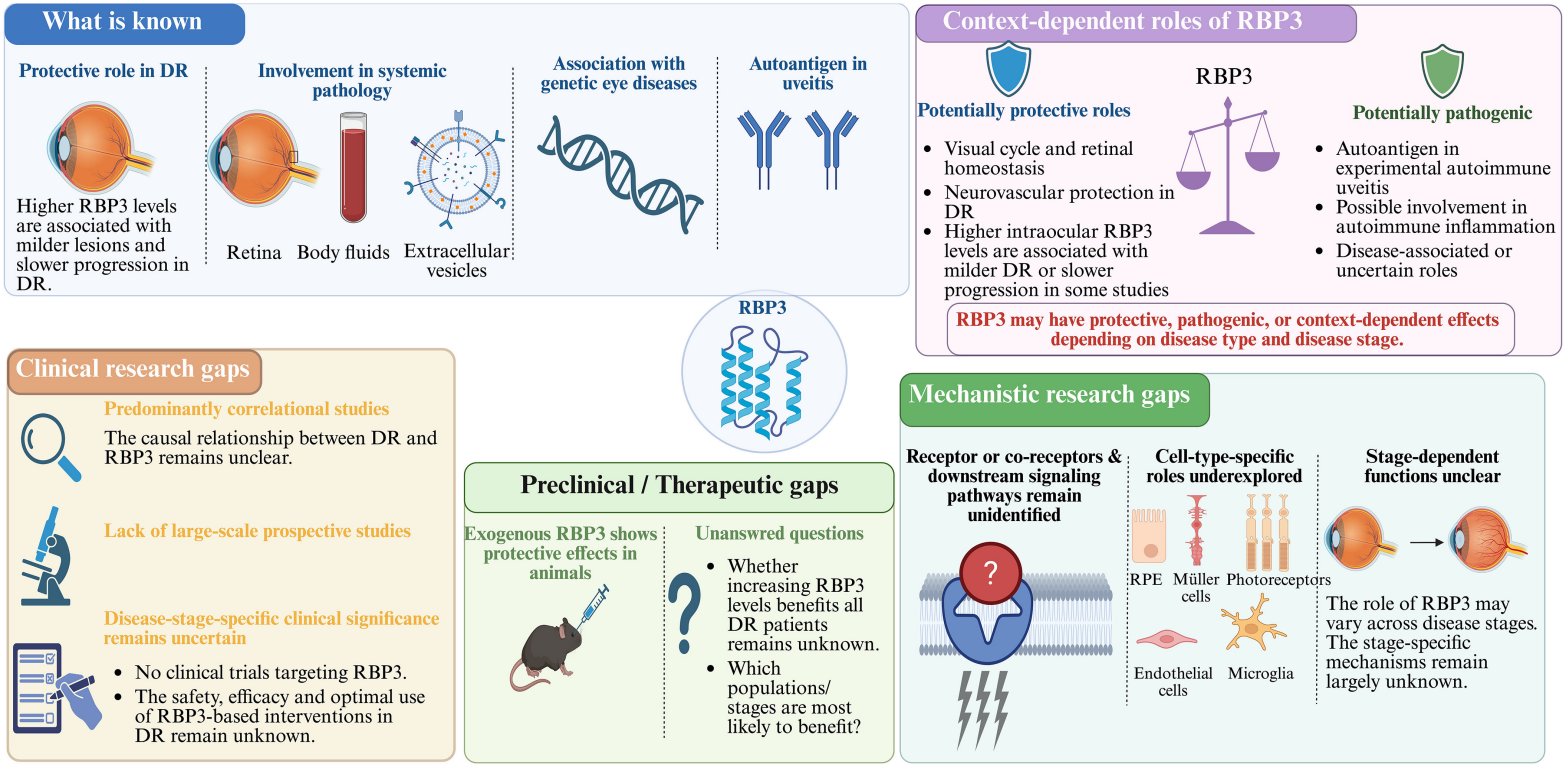
Context-dependent roles and future directions of RBP3 in ophthalmology. Current evidence suggests that RBP3 participates in the visual cycle, retinal homeostasis, and disease-related processes in several ocular disorders. In DR, higher intraocular RBP3 levels have been associated with reduced disease severity or slower progression in some studies, and exogenous RBP3 has shown protective effects in animal models. However, RBP3 may also act as an autoantigen in uveitis models, indicating that its role may be protective, pathogenic, or context-dependent depending on the disease and disease stage. Major knowledge gaps include the lack of large-scale prospective clinical studies, incomplete understanding of receptors and downstream signaling pathways, limited knowledge of cell-type-specific and stage-dependent functions, and the absence of completed RBP3-based clinical trials.

In addition, several mechanistic and translational challenges remain. The receptor-mediated actions, downstream signaling pathways, disease-stage-specific effects, and cell-type-specific functions of RBP3 are incompletely defined. The large molecular size of RBP3 also presents technical hurdles for sustained intraocular delivery or gene-based overexpression. Consequently, most current evidence is concentrated in DR, whereas studies in retinal degeneration, PM, PCG, and uveitis remain sparse. Future research should prioritize standardized detection methods, prospective clinical cohorts, mechanistic validation, and translational studies to determine the clinical utility and therapeutic potential of RBP3 across ophthalmic diseases. Current evidence and future research directions regarding RBP3 in ophthalmology are summarized in **Figure 3**.

## 7 Summary and future directions

In conclusion, RBP3 is an essential retinoid-binding protein with established physiological functions in the visual cycle and emerging disease-related roles in ophthalmology. The strongest disease-related evidence currently comes from DR, where clinical associations and preclinical studies suggest potential biomarker and therapeutic relevance (101). In retinal degeneration and PM, genetic and animal studies support a role for RBP3 in photoreceptor maintenance and ocular growth, but disease mechanisms remain incompletely defined. In PCG and uveitis, current evidence is more limited and should be interpreted as exploratory or model-dependent. Overall, RBP3 is a promising but incompletely validated biomarker and therapeutic candidate. Future studies should clarify its disease-stage-specific functions, cellular targets, receptor or co-receptor interactions, immunogenicity, safety profile, and clinical utility before RBP3-based diagnostic or therapeutic strategies can be translated into routine ophthalmic practice.

## Glossary

RBP3/IRBP: Retinol-binding protein 3/Interphotoreceptor retinoid-binding protein
IPM: Interphotoreceptor matrix
RPE: Retinal pigment epithelium
LRAT: Lecithin:retinol acyltransferase
RPE65: Retinal pigment epithelium-specific 65 kDa protein
RDH: Retinol dehydrogenase
REH: Retinyl ester hydrolase
ROS: Reactive oxygen species
CRX: Cone-Rod Homeobox
DR: Diabetic retinopathy
ERG: Recombinant human RBP3
VEGF: Vascular endothelial growth factor
VEGFR: Vascular endothelial growth factor receptor
GLUT1: Glucose transporter 1
NF-κB: Nuclear factor kappa B
RP: Retinitis pigmentosa
AMD: Age-related macular degeneration
KO: Knockout
RIP3: Receptor-interacting protein 3
PM: Pathological Myopia
GWAS: genome-wide association study
PCG: Primary congenital glaucoma
TEK: Tunica interna endothelial cell kinase
EAU: Experimental autoimmune uveitis
PTX: Pertussis toxin
sEVs: small extracellular vesicles
ADV: Adenovirus vectors
AAV: Adeno-associated virus
saRNA: Small activating RNA.
